# Editorial: Assessment of biomechanical mechanism in the context of sports injury prevention or rehabilitation

**DOI:** 10.3389/fbioe.2026.1863621

**Published:** 2026-05-07

**Authors:** Bernardo Innocenti

**Affiliations:** BEAMS Department (Bio-Electro And Mechanical Systems), Université libre de Bruxelles, Brussels, Belgium

**Keywords:** biomechanics, injury prevention, rehabilitation, Research Topic, sport injury

Sports injuries continue to represent a major challenge not only in elite and recreational sports, but also in broader societal contexts where physical activity is increasingly promoted as a fundamental pillar for health and wellbeing. With incidence rates reaching up to 10%–20%, these injuries are often associated with persistent functional limitations, delayed return to activity, and, in several cases, long-term degenerative conditions. Therefore, there is a growing need for effective prevention strategies and optimized rehabilitation approaches.

Within this context, biomechanics is a fundamental component used for understanding the complex interaction between joint kinematics, force and pressure distributions, neuromuscular control, and tissue response. The ability to quantitatively assess human motion and internal mechanical behaviour has significantly transformed both research and clinical practice, enabling a transition from qualitative to quantitative assessment, and from descriptive to predictive and, increasingly personalized approaches. Advances in motion capture technologies, wearable sensors, musculoskeletal modeling, and data-driven methods are further accelerating this transition, expanding the scope of biomechanical investigations from controlled laboratory settings to real-world applications.

The Research Topic “Assessment of Biomechanical Mechanism in the Context of Sports Injury Prevention or Rehabilitation”, published in Frontiers in Bioengineering and Biotechnology, was organized to highlight recent developments in biomechanical approaches to sports injury prevention and rehabilitation. The 20 contributions included in this Research Topic reflect the diversity of the field, spanning from joint-level mechanics, sport-specific movement analysis, neuromuscular control, clinical biomechanics, and intervention strategies.

A central theme emerging from this Research Topic is the mechanistic characterization of joint stability and injury pathways. The study by Al Kork et al. provides a comprehensive investigation of elbow dislocation through combined cadaveric testing and finite element modeling, demonstrating how flexion angle and forearm rotation critically influence joint stability (Al Kork et al.). Their findings emphasize that joint failure is a progressive, multi-structure phenomenon involving both ligamentous and bony components. At the knee joint, similar mechanistic insights are provided by Chen et al., who show that increased anterior tibial laxity leads to altered kinematics and dynamic instability during functional tasks (Chen et al., laxity study), reinforcing its role as a key risk factor for ligament injury.

Several contributions further explore dynamic, sport-specific injury mechanisms, particularly in high-demand tasks. In figure skating, Koga et al. demonstrate that increased rotational demands in jumps are associated with greater hip flexion and enhanced muscle activation, highlighting the biomechanical trade-off between performance and injury risk (Koga et al.). In badminton, He et al. reveal that unanticipated landing conditions significantly increase knee joint loading and frontal plane instability, while Xie et al. identify distinct biomechanical patterns between forehand and backhand lunges that may differentially affect ankle and knee injury risk (He et al., badminton; Xie et al.). These findings collectively underscore the importance of task specificity and unpredictability in injury assessment.

The influence of external factors such as fatigue and equipment is another key focus. The systematic review by He et al. highlights that peripheral fatigue can alter landing biomechanics, particularly increasing knee internal rotation, although results remain heterogeneous across studies (He et al., review). Complementing this, Križaj et al. show that submaximal eccentric quasi-isometric loading can maximize mechanical stimulus while limiting acute fatigue (Križaj et al.). From an equipment perspective, Xu et al. demonstrate that the geometry of carbon-fiber plates in running shoes significantly alters joint kinematics and kinetics, particularly under fatigue conditions, suggesting a direct link between footwear design and injury risk modulation (Xu et al.).

A strong emphasis across the Research Topic is placed on neuromuscular control and muscle coordination mechanisms. Studnicki et al. report that manual therapy interventions can enhance gluteus medius activation and improve balance and proprioception in athletes (Studnicki et al.), while Yin et al. show that reverse abdominal breathing in Tai Chi optimizes muscle synergy and joint stiffness, improving postural stability (Yin et al.). Similarly, Abuwarda et al. highlight phase-dependent muscle co-activation strategies in wheelchair tennis, reflecting the need for joint stability during dynamic upper-limb tasks (Abuwarda et al.). These studies collectively emphasize that neuromuscular coordination is a key determinant of both performance and injury resilience.

The role of postural control and sensory integration is further explored in multiple populations. Luo et al. demonstrate impaired balance and increased reliance on visual input in patients with cervicogenic dizziness (Luo et al., dizziness), while Miao et al. show that individuals with chronic ankle instability are particularly sensitive to visual disruption, resulting in decreased postural stability (Miao et al.). In healthy individuals, Luo et al. also identify complex interactions between stance width and visual conditions in regulating balance (Luo et al., stance width). Together, these findings highlight the importance of multisensory integration in maintaining stability and preventing injury.

Several studies address biomechanical adaptations in clinical and aging populations, reinforcing the continuum between injury and long-term dysfunction. Chen et al. demonstrate that pain associated with medial meniscus injury significantly alters gait patterns and coordination (Chen et al., meniscus), while Pan et al. show progressive biomechanical deterioration in gait with increasing severity of knee osteoarthritis (Pan et al.). In parallel, Baček et al. reveal how individuals adapt gait strategies under functional asymmetry, prioritizing spatial symmetry over kinetic symmetry to maintain stability (Baček et al.). These insights are particularly relevant for designing personalized rehabilitation protocols.

From an intervention perspective, multiple contributions evaluate rehabilitation and recovery strategies. Zhang et al. demonstrate that combined elastic band and vibration training significantly improves strength, balance, and functional performance in older adults (Zhang et al.), while Cheng et al. show that combining vibration training with kinesio taping effectively reduces pain, inflammation, and strength loss following delayed onset muscle soreness (Cheng et al.). These findings highlight the potential of multimodal interventions to enhance recovery and reduce injury risk.

Finally, the Research Topic also includes contributions addressing injury epidemiology and impact biomechanics. Bagherian et al. provide a detailed analysis of head impact characteristics in high school American football, revealing position-specific differences in impact frequency and severity, with important implications for injury prevention and equipment design (Bagherian et al.). Such studies extend biomechanical analysis beyond controlled laboratory settings into real-world scenarios.

Across all contributions, a consistent message emerges: biomechanical mechanisms underpin both injury occurrence and recovery processes, and their assessment requires an integrated, multi-scale approach combining kinematics, kinetics, neuromuscular control, and modeling. Importantly, prevention and rehabilitation should be viewed as a continuum, rather than separate domains.

Looking forward, several directions appear particularly promising. First, the integration of multi-scale approaches—linking tissue-level mechanics, joint kinematics, and whole-body motion—will be essential to fully capture injury mechanisms. Second, the increasing adoption of wearable technologies and real-time monitoring systems will allow biomechanical assessment to move beyond laboratory environments into daily life and sport-specific settings. Third, the incorporation of artificial intelligence and data-driven modeling holds the potential to improve predictive capabilities and support personalized rehabilitation pathways.

However, achieving these goals will require continued efforts toward standardization of methodologies, validation of computational models, and, crucially, closer integration between researchers, clinicians, and practitioners. Bridging this gap remains one of the most important challenges for the field.

In conclusion, this Research Topic in *Frontiers in Bioengineering and Biotechnology* highlights the critical role of biomechanical analysis in advancing sports injury prevention and rehabilitation. By combining experimental, computational, and clinical perspectives, the collected works provide valuable insights into the mechanisms underlying injury and recovery, and lay the groundwork for more precise, individualized, and effective interventions.

